# Characterization Techniques as Supporting Tools for the Interpretation of Biochar Adsorption Efficiency in Water Treatment: A Critical Review

**DOI:** 10.3390/molecules26165063

**Published:** 2021-08-20

**Authors:** Michele Castiglioni, Luca Rivoira, Irene Ingrando, Massimo Del Bubba, Maria Concetta Bruzzoniti

**Affiliations:** 1 Department of Chemistry, University of Torino, Via Pietro Giuria 7, 10125 Torino, Italy; irene.ingrando@unito.it; 2 Department of Chemistry “Ugo Schiff”, University of Florence, Via della Lastruccia 3, 50019 Sesto Fiorentino, Italy; massimo.delbubba@unifi.it

**Keywords:** biochar, activated carbon, adsorption, organic pollutants, water treatment

## Abstract

Over the past decade, biochar (BC) has received significant attention in many environmental applications, including water purification, since it is available as a low-cost by-product of the energetic valorisation of biomass. Biochar has many intrinsic characteristics, including its porous structure, which is similar to that of activated carbon (AC), which is the most widely used sorbent in water treatment. The physicochemical and performance characteristics of BCs are usually non-homogenously investigated, with several studies only evaluating limited parameters, depending on the individual perspective of the author. Within this review, we have taken an innovative approach to critically survey the methodologies that are generally used to characterize BCs and ACs to propose a comprehensive and ready-to-use database of protocols. Discussion about the parameters of chars that are usually correlated with adsorption performance in water purification is proposed, and we will also consider the physicochemical properties of pollutants (i.e., K_ow_). Uniquely, an adsorption efficiency index BC/AC is presented and discussed, which is accompanied by an economic perspective. According to our survey, non-homogeneous characterization approaches limit the understanding of the correlations between the pollutants to be removed and the physicochemical features of BCs. Moreover, the investigations of BC as an adsorption medium necessitate dedicated parallel studies to compare BC characteristics and performances with those of ACs.

## 1. Introduction

Biochar (BC) is a low-cost solid by-product of the thermal conversion of feedstocks of a different nature, such as agricultural [1], wood residues [2], manure [3], and sludge [4].

The thermal conversion procedures available at this time to obtain biochar can be grouped into the following technologies, according to the processing parameters: fast [5] and slow pyrolysis [6], gasification [7], hydrothermal [8], and flash carbonization [9]. Biochar production mainly relies on the first two technologies. Valuable information for biochars produced through pyro-gasification processes is provided by the related literature [10]. We do not aim to exhaustively discuss the biochar production technologies, regardless of how useful they are, however it is useful to recognize that each requires varying temperature range, heating rate, pressure and residence time different one to the other. Accordingly, these technologies maximize different ratios and characteristics of the final products, i.e., gas (biogas), solid (char), and liquid (tar, oil). Further, gasification (a 700–1500 °C temperature range with residence time ranging from seconds to minutes) methods mainly converts biomass to biogas, and fast pyrolysis (a 400–600 °C temperature range with residence time of seconds) maximizes bio-oil formation, whereas slow pyrolysis (a 350–800 °C temperature range with residence time ranging from seconds to hours) generally favours the formation of biochar [11].

Over the years, biochar has attracted significant attention in many fields. Indeed, it shows great potential in addressing the issue of climate change through CO_2_ storage [12] and in establishing a circular economy model [13,14] in many sectors, such as agriculture, where it could be used to regulate the carbon, phosphorus, and nitrogen cycles in soils [15]. The first applications of biochar were directed toward agricultural purposes [16] since the addition of biochar improves soil properties. In Italy, the use of biochar as a soil amendment as well as the technical requirements for this use are governed by DL 75/2010 and further modifications. The “Standardized Product Definition and Product Testing Guidelines for Biochar That Is Used in Soil” was recently released by International Biochar Initiative (IBI) to prescribe the characteristics that biochar intended for sale must possess for safe use in soil.

In recent years, studies on biochar have been extended to include water purification issues due to the adsorption properties exhibited by this material and due to its low environmental impact [17]. This new interest in biochar has led to a progressive increase of publications in this field. The first two reviews on the application of biochar for water purification, dated 2014 [11,18], followed by more recent publications [19,20], have detailed the sorption of organic/inorganic contaminants grouped by classes to elucidate possible adsorption mechanisms based on the surface chemistry of biochars. The performance of biochar towards selected classes of compounds, mainly nutrients [21], antibiotics [22], and metals [23], was the main theme of the reviews that were subsequently published on water purification using biochar.

The concept of exploiting biochar for water treatment as a potential surrogate of activated carbon (AC), the commercial adsorbent that is the most widely used in the refining tertiary stage of water treatment technologies, has escalated the need to improve the sorption characteristics of biochar. These efforts are clearly highlighted by the literature and reviews that analyse strategies for the surface modification of biochar [24] or the production of biochar with the addition of nanoscale metals [25] to the biomass before thermal conversion. However, even if BCs are proposed to possibly replace ACs in water purification, only a limited number of studies that have already been published on this topic compare the performance of BCs with those of a commercial activated carbon, thus making it extremely difficult to highlight differences between both adsorbents and the advantages or disadvantages in the use of one over the other.

Although there is established information for the use of biochars in agricultural applications, there have been no publications produced by international organizations on biochar characterization for other applications, including for water purification. Despite authors studying the removal of compounds by BCs agree with the fact that physicochemical and performance characterization is of paramount importance to understand pollutant–BC interactions [26,27], a lack of homogenous scientific approaches is observed in the current literature due to the absence of any standardization.

On this premise, it becomes relevant to compare biochar and activated carbon as adsorbents for organic micropollutants in water purification. This review addresses the way in which the two media are characterized within the literature. To the best of our knowledge, this is the first review aiming to collect and discuss the most used physicochemical and performance characterization techniques and to propose a detailed and easy-to-use compendium. In addition, the adsorption capacities of unmodified BCs and ACs toward the organic compounds investigated in the literature are compared and discussed in terms of the physicochemical properties of pollutants (i.e., K_ow_) and an efficiency BCs/ACs ratio is derived and then discussed in terms of the economic value of the two adsorbents. Moreover, the possibility of a chemometric treatment of BC adsorption capacity towards organic compounds together with the main physicochemical properties of BCs is also discussed. To the best of our knowledge, all of these aspects are innovatively investigated in this review.

## 2. Activated Carbon and Biochar: The Different Approach to Characterization

When studied for water purification purposes, it seems reasonable to compare the adsorption performance and the physicochemical features of biochar with those of activated carbon, which is by far the most widely used regulated adsorbent that is commercially available. However, the overview of the literature currently available on biochar indicates that this is not the approach followed by authors, as biochar is synthesized and tested for the removal of selected single [28,29] or mixed [30,31] compounds.

To better compare the capabilities of biochar in respect to activated carbon in water filtration, we will describe the characterization methods that are currently available for activated carbon and those that are primarily used for biochar.

### 2.1. Activated Carbon Characterization

The characterization of activated carbon is necessary in order to identify the materials that are suitable for use in the tertiary potabilization treatments or wastewater treatment plants.

The characterization of activated carbons is obtained through two core alternative approaches, which are based on the determination of (i) adsorption performance and (ii) the physicochemical parameters. The first approach determines the structure-dependent indices that are estimated in respect to standardized compounds of the proper molecular dimensions that are directly correlated to the adsorption capabilities of the activated carbon itself [32]. The second approach allows the collection of information on the main structural properties, i.e., the morphology, porosity distribution, and nature of the chemical groups present on the surface.

The adsorption performance parameters (or performance indices) of activated carbons are strictly regulated when such materials are utilized in drinking water purification processes. Many official documents, which will be briefly reviewed, specify the requirements that active carbons should meet when used for water filtration together with the methods to evaluate such performance indices. Most of these documents were released by the American Society for Testing and Materials International (ASTM), the American Water Works Association (AWWA), the German Institute for Standardization (DIN), and by the International Organization for Standardization (ISO). Thanks to the work of the European Chemical Industry Council (CEFIC), many of these methods are collected in the publication “Test Methods for Activated Carbon” [33].

Experiments to be performed on activated carbons are divided into three groups: physical tests, adsorption tests, and chemical/physicochemical tests. In the next part of this review, a brief description of the regulated parameters that need to be tested will be provided, along with the documents in which each parameter is defined. These methods are collected in Table 1.

#### 2.1.1. Physical Tests

Through physical tests, apparent or bulk density, absolute and particle density, particle size, pressure drop, and mechanical strength are determined. Apparent or bulk density (expressed as kg/m^3^ on dry basis) is defined as the mass of the unit volume of the sample in air, including both the pore system and the voids between the particles. In contrast, absolute density and particle density (also called He and Hg density, respectively) are defined as the mass of the unit volume of the solid carbon skeleton not accessible to He (for absolute density) or of the carbon particle (for particle density). Both variables are expressed as g/mL. The density parameters are necessary to evaluate the shape and size of the activated carbon particles as well as to evaluate the packing volume, bed porosity, and void fraction [46,47]. Methods to determine bulk density are described in ASTM standard [34], whereas methods to measure bulk density, absolute and particle density, particle size, pressure drop, and mechanical strength are collected by CEFIC standard [33]. Many authors agree that the monitoring of density parameters during the synthesis of activated carbons should be exploited to follow the thermal degradation of the raw precursor material [48,49].

Particle size (expressed in mm) in activated carbons influences both the adsorption of target compounds and certain mechanical properties, i.e., hydraulic conductivity and flow speed [50]. The official methods from ASTM [35] and CEFIC require the mechanical separation of the particles through sieves. It should be noted that the particle size as well as the surface morphology and porosity of the adsorbent surface [51] could also be evaluated by using physiochemical techniques, such as scanning electron microscopy (SEM) and transmission electron microscopy (TEM), as detailed in the following sections.

In addition to particle size, other characteristics such as pressure drop, resistance to flow, and mechanical strength should also be determined. The pressure drop of a gas flow over a packed bed gives information regarding a gas’ resistance to flow through the carbon layer, and the mechanical strength simulates the resistance to abrasion or friction under real conditions [36,37]. Both the particle size and mechanical strength parameters are determined for granular active carbons, which are defined by CEFIC as those having 90% of their particles larger than 0.18 mm, as determined by the aforementioned particle size test.

#### 2.1.2. Adsorption Tests

While physical tests are necessary to design drinking water filters (e.g., open gravity types), adsorption tests are required to evaluate the removal performances of activated carbons. These approaches usually combine the evaluation of the adsorption isotherm of a given adsorbate–adsorbent system and the fit to a theoretical or empirical model of the adsorption process to estimate the adsorption characteristics of activated carbons. The Freundlich model is suggested by ASTM [38,39] and CEFIC [33] to determine the adsorptive capacity of activated carbons. It can be expressed by the formula, X/M = K_F_ × C_f_^1/n^, where X/M (mg/g) is the amount of the target pollutant removed per unit mass of carbon, C_f_ (mg/L) is the residual concentration of the pollutant after treatment, and K_F_ ((mg/g)/(g/m^3^)^1/n^) and 1/n (adimensional) are the constants for a given adsorption system. The same equation is generally used in the linearized logarithmic form.

The adsorption performances of activated carbons are expressed through the determination of selected indices, namely iodine [33,40,41], phenol [33], methylene blue [33], molasses [43], and tannin [41] numbers. These parameters give information about the porous structure and the adsorption properties of the activated carbon towards target compounds with similar dimensions to the probe molecules.

To elaborate, the iodine number is specifically defined as the milligrams of iodine adsorbed from an aqueous solution by 1 g of activated carbon when the iodine concentration of the residual filtrate is 0.02 N. Procedures to evaluate this parameter have been proposed both by UNI [52] and by AWWA [37], only differing in the mathematical treatment of the results. In fact, in the UNI EN 12902 standard [52], the iodine number is extrapolated from the linear regression model obtained by plotting the mg of iodine adsorbed by three different amounts of carbons versus the residual iodine concentration, while in the AWWA B600-78 standard, the parameter is obtained by introducing a tabulated correction factor, which depends on the residual iodine normality of the filtrate.

When comparing both of these protocols to calculate the iodine number, the AWWA B600-78 standard is both less time consuming and less laborious. However, the correction factor is derived according to the residual concentrations typically obtained by activated carbons. This implies that, as a general consideration, the method cannot be applied to materials with adsorbing capacities that are substantially different to those of activated carbon since the residual iodine concentrations would be outside the range of those specified by AWWA. UNI EN 12902, alternately, does not require any mathematical treatment and has the advantage of being applicable to a wider range of adsorbent materials of different capacities. However, as UNI EN 12902 does require the determination of an adsorption isotherm, this approach is obviously more time consuming than that described by the AWWA method that was previously mentioned.

The phenol number is defined as the phenol adsorption for a singular weight unit of carbon when, after adsorption, the phenol concentration in a solution decreases from 10 to 1 mg/L.

The methylene blue index—defined as the methylene blue volume (in mL) of a 1.2 g/L solution adsorbed by a known amount of sorbent (0.1 g) within a prearranged contact time (5 min)—was demonstrated to be directly correlated to the specific surface area (SSA) and micropore volume of the adsorbent [53]. Official procedures to evaluate the aforementioned indices have been detailed by the ASTM and the Water Research Commission (WRC) [54,55].

The iodine and phenol indices are commonly related to the presence of micropores (<2 nm), and they are therefore considered to be informative of the effectiveness in removing small-sized organic water pollutants. On the other hand, the methylene blue index is linked to the abundance of mesopores (2–50 nm) and thus is a useful indicator of the adsorption capacities for medium–large-sized organic pollutants [56].

The tannin number is defined as the concentration of activated carbon (mg/L) that is required to reduce the concentration of a standard tannic acid solution from 20 to 2 mg/L. Tannins, a mix of medium- and large-sized molecules, can be efficiently adsorbed by the mesopores and macropores (>50 nm) of the activated carbons. The tannin values of different activated carbon substrates and the pore volume of mesopores are linearly correlated [57], and carbons with low tannin numbers exhibit the highest quality of removing high molecular weight impurities.

The molasses number is a measure of the degree of decolorization of a standard molasses solution that has been diluted and standardized against standardized activated carbon. The molasses number represents the potential pore volume that is available for larger adsorbing species.

Of these adsorption tests, the iodine index is the only one required by the UNI EN 12915-1 standard for products used for the treatment of water intended for human consumption and for which a specific requirement is set (>600 mg/g).

An additional adsorption test for activated carbons, also collected in the CEFIC report, is the phenazone index. Since it is not directly linked to the removal of pollutants from waters but is instead linked to pharmaceutical purposes [58,59,60], the phenazone index will not be further detailed.

#### 2.1.3. Chemical and Physicochemical Tests

Chemical tests are expected when activated carbon must be used for drinking water filtration in potabilization plants, as they are utilised to assess the purity criteria for adsorbents. As an example, according to the EN 12915-1 standard [40], ashes, water-soluble material, and water-extractable substances (As, Cd, Cr, Hg, Ni, Pb, Sb, Se, CN-, fluoranthene, benzo[b]fluoranthene, benzo[k]fluoranthene, benzo[a]pyrene, benzo[g,h,i]perylene, indeno[1,2,3-cd]pyrene) must be evaluated for activated carbons. These substances represent the most probable impurities that may be present in the adsorbent as a result of both raw material composition [61] and thermal process conditions [62]. Limits are provided for these parameters. As far as the determination of water-extractable substances is concerned, extraction is performed in a solution containing sodium hydrogen carbonate, calcium chloride, and magnesium sulphate [40,52]. It is important to underline that users should be notified of the presence of other impurities not included in the standard should be, as defined by the same standard. Notably, the determination of the aforementioned polycyclic aromatic hydrocarbons (PAHs) and metal content in activated carbon is foreseen by the CEFIC procedure [33] under more drastic conditions, such as cyclohexane extraction and total oxidation, respectively.

Strictly related to the operating procedures and start-up operations of the refining stage inside the plant is the pH value of the solutions in contact with the adsorbent. The pH conditions strongly influence the duration of the washing procedures of the carbon before it reaches its full operability. Inorganic and chemically active groups on the carbon surface are responsible for the possible modification of the pH when the substrate is placed in contact with water. Among the procedures available for the determination of this parameter, the ASTM D6851-02 and D3838-05 standards [44,45] consider the contact pH and the pH of a boiled water extract, respectively, in a 1:10 *w/v* ratio.

In addition to the techniques detailing the characterization of activated carbons that have been presented above, the literature that is currently available uses other techniques and approaches with the aim of fully understanding surface and bulk chemistry and of speculating on possible interactions with the target compounds. As an example, the evaluation of the pH of zero-point charge (PZC), the pH value at which the net charge density of the material is equal to zero, could be important to define the adsorption behaviour of a material towards pollutants of different charges at different pH conditions [63].

Additional information could also be obtained through Boehm’s titration, by which the equivalent of surface acidic/basic functionalities (carboxyl, lactone, phenolic groups) per gram of carbon can be obtained [64]. This approach gives deeper insight into surface chemistry.

Directly connected to the surface charge of the material, the cationic exchange capacity (CEC) is defined as the total amount of the exchangeable cations of a sorbent [65,66]. Microscopy (both scanning and transmitting, SEM and TEM), nitrogen adsorption isotherms, and Fourier transform infrared spectroscopy (FTIR) are usually applied in order to derive a topographical morphology of the material and to define the particle dimension and porous structure as well as to study the surface functional groups and structure [67].

An additional characterization of activated carbons is based on the analysis of the elemental composition. From the amount of carbon, oxygen, and hydrogen, it is possible to derive the Van Krevelen diagram, in which the O/C ratio is plotted against the H/C ratio [68]. The resultant graph is used to classify carbons and their feedstock [69] and to fully understand the evolution of carbon during the heating treatment, thus predicting specific the properties of carbon adsorbents (degree of carbonization by H/C ratio, hydrophilicity by O/C ratio, polarity by (O + N)/C ratio) [70].

### 2.2. Biochar Characterization

#### 2.2.1. Chemical and Physicochemical Tests

At this time, no official method for the evaluation of the performance and characteristics of biochars intended for water filtration has been published. It is of the authors’ opinion that, in the absence of indications by regulatory bodies, official procedures previously presented for activated carbons (EN, CEFIC, AWWA standards, see Table 1) should be homogenously applied to test the performance of biochars. This approach would ensure the coherence of the results presented in the literature and the straightforward comparison among the biochars produced and a comparison between biochar and activated carbon.

Studies dealing with the removal of contaminants from water using biochar show that biochar is synthesized from different types of feedstock and different thermal process conditions. The extent of adsorption of different pollutants is generally correlated with certain properties of the biochar that are specifically measured to characterize the biochar produced.

Table 2 collects the main approaches and instrumental techniques used for the characterization of biochar according to the literature of the last decade concerning the removal of organic pollutants using biochar. The following are indicative, though not exhaustive, examples of the characterization of biochars that have been tested for water filtration.

Many authors [71,72] apply nitrogen adsorption isotherms to derive information about surface area, pore volume, and average pore size. We have previously pointed out that these parameters are clearly correlated with the widely discussed adsorption indices for activated carbons, which have rarely been determined for biochar. A thorough search of the relevant literature yielded few studies presenting this correlation for biochar [56,73], which is in contrasty to what has been presented for activated carbon [32,74,75]. Of note, recent literature now measures the adsorption indices, i.e., the iodine index, as an indicator of the best adsorption performance of biochars obtained under different processing conditions (e.g., heating rate). The iodine and methylene blue indices have been proposed as comparative indicators for the performance of activated carbons and biochars [76].

The physicochemical methods used for the characterization of biochars mainly depend on the objectives of the study in question. However, these methods are used to define the surface properties of biochars and to highlight possible adsorption mechanisms that are responsible for biochar–pollutant affinity [71,77]. Among these methods, FTIR, XPS, and XRD are the most frequently applied to characterize surface chemistry.

FTIR spectroscopy is a widespread technique used to investigate the surface chemical functional groups (e.g., aliphatic or aromatic nature) in biochar [78]. Through the FTIR spectrum, vibration bands can be assigned to defined functional groups that enable speculation on possible interaction mechanisms with the pollutants of interest [11]. Additional information gained from FTIR is the understanding of the reactions that occurred during thermal treatment steps [70,79,80].

XPS analysis provides information regarding the chemical composition and bonds on the biochar surface to a surface depth < 10 nm and on the relative abundance of different species of certain elements on the surface, e.g., different C and N containing groups and bonds (C-C, C-H, C=O, -COOH, N-C, amino acid N, and ammonium-N) on the biochar surface [81,82].

The structure and phase composition of biochars can be derived by XRD analysis. The information that is gained can be important for determining if the successful preparation of modified biochar occurred [77] or to understand the evolution of the thermal process with respect to the initial biomass composition [83].

Van Krevelen plots, which were previously discussed in the activated carbon section, are extensively employed in biochar characterization to define biochar composition as a function of the thermal conditions used for biochar production [70,84,85].

**Table 2:** Main characterization methods employed for biochar according to the recent literature.

Test	Ref Biochar
**Adsorption Indices**	
Iodine number	[56,76,86,87,88]
Methylene Blue number	[56,76,89]
**Chemical Tests**	
Total PAHs	[90]
pH, ash	[56,89]
**Surface and Functional Groups Characterization**	
Nitrogen-adsorption isotherm	[30,56,71,91,92]
FTIR	[56,89,92,93,94]
SEM	[92,94,95]
XRD	[56,92,93,95]
Zero-point charge	[56,93,94,96]
Boehm’s titration	[89]
Cation exchange capacity	[89]
**Elemental Composition**	
H/C, O/C, (O + N)/C (Van Krevelen plots)	[56,84,85,95]
Kinetic and isotherm studies	[56,97,98,99,100]

#### 2.2.2. Adsorption Tests

The careful study of the current literature provides strong indications that the assessment of the adsorption features of biochars is mainly performed and that the studies in which the adsorption performance of the biochars are simultaneously compared with that of activated carbon are extremely limited. This approach makes understanding the benefits or limitations of using biochar in place of activated carbon incredibly complex. In this regard, it is of the authors’ opinion that research on the use of biochar for water refining should be addressed a priori by always including activated carbon for a direct comparison.

Materials intended for the removal of pollutants from waters are usually described with reference to their retention performance through kinetics and adsorption isotherms [101].

The optimization of the contact time is essential for further adsorption studies to ensure the presence of equilibrium conditions within pollutant-adsorbent (biochar/activated carbon) systems. Kinetic studies have also provided additional information, including the identification of rate controlling steps such as (i) the transport of the solute molecules from the aqueous phase to the surface of the solid (film or external diffusion); (ii) the transfer of the solutes from the surface to the intra-particle sites (intra-particle diffusion); (iii) and the adsorption of the solutes on the interior surfaces of the adsorbent [102]. Pseudo-first order and pseudo-second order models are the most frequently computed models. However, there are some less common works that thoroughly investigate the adsorption performances of BCs through the Elovich model [103] (predicting the mass and surface diffusion and the activation/deactivation energy of the system) or through the intraparticle diffusion and Boyd models [104,105] (which determine the effect of internal and external diffusion on the adsorption mechanism).

Once the equilibrium conditions are identified, adsorption isotherm data are obtained to describe the type of interactions involved between adsorbates and adsorbents and to quantify the extent of the adsorption (i.e., the adsorbent capacity). The correlation of the isotherm data using theoretical or empirical equations is useful for practical operation. In fact, a proper understanding and interpretation of adsorption isotherms is crucial for the overall improvement of the adsorption mechanism pathways and the effective design of an adsorption system [106]. Because of its wide applicability, linear regression analysis is frequently used to fit experimental data and to assess adsorption performance in the biochar related literature [107,108]. The Langmuir, Freundlich, and Tempkin models are the most applied models [97,98,99,109,110,111,112,113], with limited works also investigating the Dubinin-Radushkevich [114] and Sips [115] models. Non-linear regression analysis [116], which has also been widely used by a number of researchers in an attempt to minimize the gap between the predicted and experimental data, is also applied to biochar studies [117]. The description of the aforementioned models [118,119] is out of the scope of the present review.

Our survey showed that recent publications on adsorption onto BCs dedicate significant effort to the study of adsorption kinetics and isotherms. In contrast, simultaneous studies on both BCs and ACs are only rarely considered for comparison [56,99,120], resulting in a consequent lack of information.

#### 2.2.3. Adsorption Tests in Dynamic Bench Scale and Pilot Scale Conditions

In addition to what has previously been presented, where the adsorption performances of BCs were reported for batch conditions, the evaluation of kinetic and isotherm models in dynamic conditions by bench scale and pilot scale systems is of paramount importance in order to simulate real BC applications in filtering systems and to achieve technology readiness levels (TRL) that are higher than an experimental proof of concept.

Despite this premise, few studies evaluate the retention of pollutants from water matrices on unmodified BCs using small lab-scaled columns [121], and even fewer studies investigate the adsorption isotherms [122]

In dynamic testing conditions, kinetic models should be replaced by breakthrough curves, in which the profile of the effluent adsorptive concentration is continuously measured at the outlet of a fixed bed adsorber. It should be noted that these curves can be considered as the last of the essential characterizations of an activated carbon since they simulate performance in an industrial application [123]. Whilst it seems reasonable to adopt the same curves to evaluate the performances of BCs as adsorbents for aqueous solutions, most studies work at laboratory scale conditions [124]. This is most likely due to an undeveloped biochar technology.

When considering pilot or pre-commercial scaled systems, in which higher volumes of water are treated using biochar packed columns, the calculation of adsorption performance is only obtained through the transposition of isotherm models from batch tests to pilot scale conditions [121].

Finally, it is interesting to highlight a lack of adsorption performance studies in bench- or pilot-scaled conditions towards organic pollutants, which is the object of our review. In comparison, greater attention is devoted to the adsorption of metal ions [125,126,127,128]. It is therefore evident that further research and insights are necessary for TRL improvement.

#### 2.2.4. Leaching Tests

As previously described for ACs (Section 2.1.3), leaching tests for organic (PAHs) and inorganic compounds (metals) are necessary to evaluate the safety of the supports employed for the removal of pollutants in water treatment [52]. Therefore, for BCs that are intended to be used for water filtration, considering that these adsorbents are produced by thermal treatment similar to ACs, then the same characterizations should be suggested. Despite this assumption, few papers have evaluated the leaching of some classes of compounds in aqueous medium from biochars studied for water treatment [56,129]. Specifically, Hong and co-workers [129] extensively describe the release of selected heavy metals from biochar in ultrapure water together with nutrients and total organic carbon as representative indicators of organic matter without focusing on any specific class of compounds. In a pioneering vision, Del Bubba and co-workers [56] followed the analytical protocols imposed by UNI EN 12915 [40] for evaluating the leaching characteristics of metals and PAHs while comparing BC vs. AC performance.

Apart from the limited works that have been previously noted, the actual literature shows a disproportionate number of papers exploring the use of leaching tests in BCs used as an amendment in agricultural applications [130,131] over water treatment. This highlights the different maturity stages of BCs used in agriculture compared to those used in water treatment.

## 3. Biochar as Adsorbent for Organic Micropollutants

### 3.1. Typical Target Pollutants and Performance Capabilities

The application of biochar as an adsorbent for the removal of organic pollutants from water and wastewater has been extensively studied. A search of the Web of Science database for the words “biochar” and “adsorption” yields approximately 5800 studies, with about being 40% focused on the removal of heavy metals (research refinement using “metal”) and the other 40% being focused on organic compounds (research refinement using both “organic” and “pollutants”). However, despite the volume, a critical approach investigating the possible correlation between biochar adsorption capacities and both the intrinsic properties of target compounds (i.e., polar character) and/or the physicochemical features through statistic tools is missing. Therefore, the following paragraphs will focus on a critical investigation on this topic, limiting the discussion to the literature of the last ten to fifteen years relating to the removal of organic pollutants by unmodified biochars. As previously mentioned, since activated carbon is the standard material for water treatment, ACs will be considered in parallel.

From a subset of this literature, a representative list of the organic compounds that are the most frequently removed by biochars was retrieved and summarized in Table 3. Papers were only selected if adsorption capacity data at equilibrium conditions were presented. Each analyte is clustered depending on its ionic form (undissociated, anionic or cationic) at a neutral pH value, which is typical of raw wastewater. The logK_ow_ and the pk_a_ (if available) values are reported. Within each reviewed paper the comparison of BC performances with those of AC is reported if present.

The data collected in Table 3 allow several considerations that will be discussed.

Most of the organic pollutants tested for removal by biochars are dyes, herbicides, or drugs, as they account for the most detected species in wastewater.

Regarding the ionizability properties, the reported data clearly show that at the pH value typical for wastewater and natural water (pH ~ 7), most of the organic molecules that are typically investigated are present in neutral form. However, it should be noted that, among the neutral compounds that are considered, many of them are ionizable species with pKa values higher than 7, thus suggesting that the chemistry of their functional groups will be altered at different pH conditions with consequent variation in the adsorption mechanism [164] and in BC removal performance.

**Figure 1:**
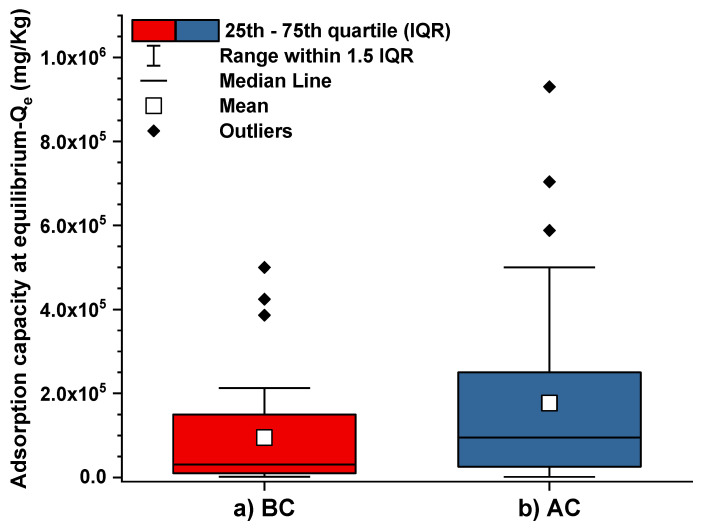
Boxplot of adsorption capacities at equilibrium for BCs (**a**) and ACs (**b**). *n* = 50 for both series. Inter quartile range (IQR), median, means, and ranges were calculated through OriginLab software. Ranges were calculated as 1.5-fold the IQR, according to Apton and Cook [165].

**Figure 2:**
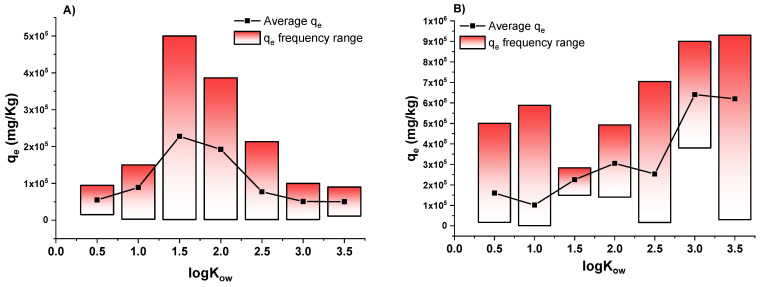
Distribution plot of equilibrium adsorption capacities (q_e_, mg/kg) as a function of the logK_ow_ of organic target pollutants removed by biochars (**A**) and activated carbons (**B**). *n* = 50 for both adsorbents. The clusters considered the log K_ow_ range from 0.5 to 3.5.

The analysis of the reviewed literature shows that most of the studies do not consider ACs in their investigations, limiting the discussion to the selected BCs. This approach makes it difficult to assess BC performance and its advantages over more traditional adsorbents, i.e., AC. Therefore, to understand and to critically discuss BC capabilities compared to AC, the range of the typical adsorption capacities of BCs and ACs towards the most studied organic pollutants was derived through a distributional statistical approach. The maximum adsorption capacity at equilibrium conditions (q_e_ expressed as mg/kg) derived from data from adsorption isotherm tests (see Section 2.2.2) was chosen as a representative parameter for adsorption efficiency. Statistical treatment was not limited to the data in Table 3 (restricted to the last 10–15 years of literature) but was extended to less recent literature to achieve a representative population of 50 studies. The collected q_e_ data for BCs were treated using a boxplot tool (Figure 1a) that enabled an evaluation of the data dispersion and the main statistical indices (i.e., average, median, etc). In a similar manner, a representative data population of q_e_ for ACs was reported (Figure 2b, *n* = 50 as for BCs). It is important to highlight that due to the scarcity of studies that simultaneously include BCs and ACs, to achieve the same data numerosity (*n* = 50), it was necessary to also include studies that were only related to ACs.

Graphical results demonstrate that the inter quartile range (IQR-green box), which is between 25th (Q1) and 75th quartile (Q3), obtained for the q_e_ values of ACs is wider than the one obtained for BCs. Indeed, for BCs, the Q1 and Q3 values are 10,000 mg/kg and 150,000 mg/kg, respectively, with an IQR area (Q3-Q1) of 140,000 mg/kg, while for ACs, the Q1 and Q3 values are 21,400 mg/kg and 266,400 mg/kg, respectively, with an IQR area of 245,000 mg/kg. These data clearly highlight that activated carbons show a wider range of adsorption capacities in respect to BCs of about 50% and higher q_e_ values. A similar gap is also observed when considering the average capacity (95,000 mg/kg and 176,800 mg/kg for BCs and ACs, respectively).

As proposed in the Introduction section, these observations support of the need to increase research on properly modified BCs.

### 3.2. Effect of Hydrophobic Character of Analytes on Biochar Adsorption Efficiencies

When considering the intrinsic properties of the compounds under investigation, one of the most important elements to be accounted for is the hydrophobic/hydrophilic characteristics, since they strongly influence the type and the strength of the interaction occurring between the biochar surface groups and the target pollutants. Therefore, to produce evidence of a possible correlation trend between the adsorption capacity of BCs and the hydrophobic characteristics of the organic compounds that are being removed, a distribution graph was chosen. For this purpose, the distribution of BC q_e_ (mg/kg) data taken from the literature (*n* = 50) was plotted as a function of the logK_ow_ values of the target pollutants to be removed (Figure 2A). It should be specified that, from all of the data that was previously retrieved (Table 3), only the molecules having a logK_ow_ ranging from 0.5 (±0.25) to 3.5 (±0.25) were chosen for statistical treatment since those outside of those values have a reduced frequency, rendering their processing insignificant. The logK_ow_ values of the selected target compounds are referred to slightly hydrophobic to medium-high hydrophobic analytes.

The same distribution was also represented for the same molecules adsorbed on activated carbons (*n* = 50) in Figure 2.

To elaborate, in both graphs (Figure 2A,B), the bars represent the min–max q_e_ frequency range for each 0.5 unit of the logK_ow_ cluster (±0.25) in which all of the target compounds were divided. The black squares represent the average q_e_, and the black lines represent the average q_e_ trend line.

The data representation in Figure 2 leads to interesting observations. When focusing on the average q_e_ values, a Gaussian-similar trend could be observed for BCs (Figure 2A). In fact, molecules characterized by logK_ow_ ranging from 1.5 to 2 are more retained from BCs than those characterized by lower and higher hydrophobic characteristics. Conversely, similar behaviour is not observed when studying the removal of the same compounds using ACs (Figure 2B) since adsorption increases with the increase of logK_ow_, reaching a plateau at logK_ow_ that is approximately equal to 3.0 (medium–high hydrophobicity).

Such results could be explained by examining the different interactions between the organic pollutants and the biochars or activated carbons as a result of the different surface properties of the two adsorbents. In fact, it can be determined that the surface of ACs is characterized by a higher degree of aromaticity with respect to the surface of BCs since their production temperature and activation processes are demonstrated to convert aliphatic carbons into aromatic carbons [164,166]. This feature is reflected in the preferred π-π and/or hydrophobic interactions in ACs [167] rather than BCs. Consequently, the higher the aromaticity of the molecule and the higher the hydrophobicity of the target pollutants, the stronger their interactions, and, therefore, the higher the adsorption capacities of the activated carbons will be.

Alternatively, due to their typical process production conditions, BC is characterized by a surface containing both aliphatic ionizable moieties (i.e., -COOH), and, to a lesser extent, aromatic carbons. In fact, the lower pyrolysis temperature conditions in BC production reduce the conversion of the aliphatic C to aromatic C, so the H/C ratio is typically higher in biochars [168] than in activated carbons [169]. Therefore, a wider range of mechanisms, such as electrostatic, π-π/hydrophobic, and hydrogen bond interactions, are involved within the pollutant and the BC surface [164]. Consequently, organic pollutants characterized by a medium range hydrophobic character, i.e., logK_ow_ of about 1.5–2 (Figure 2B, which displays both polar and nonpolar functional groups) are expected to interact with the biochar surface, synergically exploiting all of the previously mentioned mechanisms. Conversely, target compounds with lower logK_ow_ values are expected to interact with BC via a reduced number of interactions (electrostatic, if ionizable, or hydrogen bond interactions), thus resulting in lower adsorption on BCs. In contrast, pollutants with higher logK_ow_ values are expected to interact with BC via π-π or hydrophobic interactions, which only limit the extent of retention.

The distribution plots reported here show that the use of biochar in water filtration and the activated carbon replacement could be suggested for the removal of medium non-polar compounds (i.e., herbicides), for which the affinity is similar to activated carbons (average q_e_ about 2.5 × 10^5^ mg/kg for both sorbents). In this regard, the use of highly energy demanding activation processes typical of ACs could be avoided.

## 4. Considerations on the Correlations of Physicochemical and Performance Indices with Biochar Adsorption Efficiencies through Chemometric Approaches

As described in Section 2 of this review, several physicochemical and performance indices of biochars strictly influence adsorption performances. Selected authors have tried to correlate only one biochar property at a time (i.e., pyrolysis temperature [6], feedstock [170], etc.) with the adsorption efficiencies. Nevertheless, an overall evaluation that considers the broadest possible physicochemical parameters and indices is still missing. A principal component analysis (PCA), a chemometric approach frequently applied to directly highlight the correlation status of several variables, would be highly desirable to evaluate the possible correlation between the main biochar features described in Section 2.1 and Section 2.2 and the adsorption capabilities expressed, for instance, as the maximum adsorption capacities derived from the Langmuir models (see Section 2.2.2) or as the K_F_ derived from the Freundlich models (see Section 2.1.2). Despite the volume of papers devoted to the removal of organic compounds by biochars, a lack of homogenous data on physicochemical and performance characterization is observed since most of the studies only evaluate a few of the parameters that were previously mentioned (Section 2) depending on each author’s particular requirement. For this reason, when trying to set-up a PCA matrix by retrieving data from the available literature, the absence for relevant data makes chemometric treatment impossible [171]. To overcome this obstacle, a possible solution to perform chemometric PCA treatment could be to either work with the data from a single study, in which, for example several biochars are tested, or to reduce the variables to be computed to those few that are in common among the selected works. However, in both cases, the number of treated samples or variables to be considered would be so reduced that significant results from PCA could not be obtained.

## 5. Economic Evaluation on Biochar Use over Activated Carbon

For the compounds included in this review, the consideration reported in Section 3.1 showed that the adsorption capacities for ACs are superior to BCs. However, removal effectiveness is not the only parameter to consider when evaluating the applicability of adsorbents in wastewater treatment. Production costs should also be considered.

We have derived an effectiveness/cost index, weighting the adsorption performances with the production costs, evaluated according to the literature survey. To further elaborate, it has been recently reported that the AC production costs range from 1.35 (China) [172] to 2.6 EUR/Kg (Italy) [56]. For BCs, these values range from 0.15 (Italy) [56] to 0.42 EUR/Kg (U.S.) [173,174], with average values of 1.98 EUR/Kg and 0.29 EUR/Kg, respectively. On the basis of the mean q_e_ values discussed above in Section 3.1, (95,000 mg/kg and 176,800 mg/kg for BCs and ACs, respectively), an effectiveness/cost index has been calculated for both BCs and ACs, dividing the average maximum equilibrium adsorption capacity (q_e_, expressed as mg/kg) by the average cost per Kg of adsorbent. The results (3.3 × 10^5^ mg removed for each EUR of BC and 8.9 × 10^4^ mg/EUR for ACs) show that although BCs have lower adsorption capabilities, their effectiveness/cost index is 3.7 times higher than that of activated carbons, which means that for the same capital investment, a removal of organic pollutants that is approximately 4-fold higher (in terms of mg) is obtained using BCs compared to ACs.

## 6. Conclusions

In the last ten–fifteen years, biochars intended for water remediation have become a trending topic, with thousands of published works studying their possible application for the removal of both organic and inorganic contaminants. The data reviewed and depicted using boxplot statistics indicate that BCs exhibit slightly lower adsorption capacities for organic compounds, especially for those of medium–high hydrophobicity, in comparison with standard adsorption materials (activated carbons). Despite the reduced adsorption capacities of BCs, the production cost is significantly lower, with a calculated effectiveness/cost index that is about 4 times higher for BCs in respect to ACs.

BC removal efficiencies could be correlated with the main properties of the target compounds, such as the hydrophobic characteristics as expressed by the logK_ow_ values. According to our survey, for medium non-polar compounds, the replacement of ACs with BCs in tertiary treatments appears feasible since average BC adsorption efficiencies are in the same range as those of AC. This finding supports the implementation of column experiments for establishing the maximum loading capacity of BCs in experimental conditions that are more similar to a real-life scale.

The correlation of the intrinsic physicochemical and performance parameters with biochar removal capacity is more challenging. The presented review highlighted a lack of homogenous physicochemical and performance characterization techniques, which, in our opinion, are mandatory for an overall detailed understanding of the core interactions and correlations between the pollutants and the BC surface. A correlation of this type is absent in the current literature.

The comprehensive survey on the activated carbon characterization protocols (most of them proposed by regulatory organisations), as reviewed in the first part of this paper, proves that by using these procedures, a full understanding of the capability and safe use of ACs in the removal of pollutants from water can be achieved. It is consequently desirable that, because of the lack of regulatory methods specific to biochars, all protocols for ACs should be also homogenously applied for the characterization of BCs intended for water purification, thereby avoiding fragmentary and subjective characterization that only depends on the predilection of the authors. This approach would allow the verification of whether the biochars prepared in the various experimental conditions comply with the requirements set out in the aforementioned standards relating to the absorbent materials intended to be used for the filtration of drinking water. A direct comparison between the capabilities of BCs and ACs is also recommended to explore the scale up of BC applications (bench and pilot scales) towards higher TRL levels.

Additionally, through this overview, an easy-to-use compendium on the main physicochemical and performance characterization techniques of both BCs and ACs is provided.

Finally, the currently available literature data for BCs are non-homogeneous which makes it impossible to extract detailed information through the main chemometric tools, such as PCA. Developing a body of knowledge of the main physicochemical and performance characteristics of the BC that is as complete as possible is therefore of fundamental importance to statistically evaluate the possible correlations between the removal capacity of BCs and intrinsic BC physicochemical properties.

## Figures and Tables

**Table 1:** Standard methods for the characterization of activated carbon.

Test	Method	Reference
**Physical Tests**		
Bulk density	ASTM D2854, CEFIC	[33,34]
Absolute density	CEFIC	[33]
Particle density	CEFIC	[33]
Particle size	ASTM D2862, CEFIC	[33,35]
Pressure drop	CEFIC	[33]
Mechanical strength	ASTM D3802, AWWA B604, CEFIC	[33,36,37]
**Adsorption Tests and Indices**		
Adsorption isotherm	ASTM: D3860-98, 5919-96, CEFIC	[33,38,39]
Iodine number	AWWA B600-16, EN 12915-1, ASTM D4607-14, CEFIC	[33,40,41,42]
Phenol number	CEFIC	[33]
Methylene Blue number	CEFIC	[33]
Molasses number	EPA625171002A	[43]
Tannin number	AWWA B600	[41]
**Chemical Tests**		
Ashes, water soluble material, and water-extractable substances (As, Cd, Cr, Hg, Ni, Pb, Sb, Se, CN-, fluoranthene, benzo[b]fluoranthene, benzo[k]fluoranthene, benzo[a]pyrene, benzo[g,h,i]perylene, indeno[1,2,3-cd]pyrene)	EN 12915-1	[40]
pH	ASTM: D6851-02, D3838-05	[44,45]

**Table 3:** List of the most investigated organic pollutants removed by biochars in the last ten years’ worth of literature. Compounds are grouped following their ionizable properties. The values of their logK_ow_ and pK_a_ (if applicable), as calculated using Chemicalize Software [132], are reported.

**CATION**	**Class**	**logK_ow_**	**pK_a_ (Ionic Group)**	**Comparison with AC**	**Ref**
Methylene blue	Dye	0.75	/	No	[133,134,135,136,137,138]
Methyl violet	Dye	0.43	9.17	No	[139]
Malachite green	Dye	0.8	/	No	[140]
Lincomycin	Antibiotic	−0.3	7.97	No	[141]
**ANION**	**Class**	**logK_ow_**	**pK_a_ (Ionic Group)**	**Comparison with AC**	**Ref**
Sulphapyridine	Antibiotic	0.35	6.24	Yes	[142,143]
Sulfamethoxazole	Antibiotic	0.79	6.16	No	[144]
p-coumaric acid	Drug	1.46	3.81	No	[145]
Reactive brilliant blue	Dye	−1.33	−2.69	No	[146]
Congo Red	Dye	2.63	/	No	[135,147,148,149]
tris(2-carboxyethyl)phosphine	Flame retardant	1.78	3.22; 4.38	No	[121]
2,4-dichlrophenenoxiacetic acid	Herbicide/Pesticide	2.61	2.81	Yes	[150]
t-Cinnamic acid	Precursor	2.13	4.32	No	[145]
**NEUTRAL**	**Class**	**logK_ow_**	**pK_a_ (Ionic Group)**	**Comparison with AC**	**Ref**
Amoxicillin	Antibiotic	−2.3	7.22	No	[151]
Tetracycline	Antibiotic	−3.4	7.36	No	[22,152,153]
Ciprofloxacin	Antibiotic	−0.8	5.56; 8.77	No	[154]
Sulfadiazine	Antibiotic	0.38	7	No	[22]
Chlortetracycline	Antibiotic	−1.98	2.99	No	[155]
1H-benzotriazole	Corrosion Inhibitor	1.44	9.04	No	[121]
p-nitrotoluene	Dye	2.37	/	No	[156]
Bisphenol-a	Endocrine disruptors	3.32	9.78; 10.39	No	[157]
Atrazine	Herbicide	2.61	/	No/Yes	[121,158]
Diuron	Herbicide	2.68	13.18	No	[121]
1-naphtol	Herbicide	2.85	9.6	Yes	[159]
Catechol	Herbicide	0.9	9.34; 12.79	No	[160]
Carbaryl	Herbicide	0.9	/	No	[161]
17α-ethinyl estradiol	Estrogen	3.67	10.33	No	[157]
Phenol	Plastic production	1.46	10.02	No	[162]
Phenanthrene	Polycyclic Aromatic Hydrocarbon	3.71	/	No	[157,163]
Naphthalene	Polycyclic Aromatic Hydrocarbon	2.96	/	No/Yes/No	[156,159,163]
Trichloroethylene	Solvent	2.42	/	Yes	[6]
4-tert-Octylphenol	Non-ionic Surfactant	5.18	10.23	Yes	[56]
TRITON^TM^X-45 (mixture of 4-t-octylphenol polyethoxylated)	Non-ionic Surfactant	3.49–4.90	15.10 ^a^	Yes	[56]
4-(1-Ethyl-1,4-dimethylpentyl)-phenol	Surfactant	5.79	10.22	Yes	[56]
IGEPAL^®^CO-520 (mixture of branched 4-nonylphenol polyethoxylated oligomers)	Surfactant	4.26–5.50	/	Yes	[56]

^a^ Referred to the compound 2-[4-(2,4,4-trimethylpentan-2-yl)phenoxy]ethan-1-ol, as retrieved from Chemicalize.

## Data Availability

Not applicable.
